# Study on a Pseudo-Elastic Model for High-Damping Rubber

**DOI:** 10.3390/polym16213042

**Published:** 2024-10-29

**Authors:** Zhihao Guo, Tianbo Peng

**Affiliations:** 1 College of Civil Engineering, Tongji University, Shanghai 200092, China; guozhihao0103@foxmail.com; 2 State Key Laboratory of Disaster Reduction in Civil Engineering, Tongji University, Shanghai 200092, China

**Keywords:** high-damping rubber, pseudo-elasticity theory, multiple influencing factors, Mullins effect, stiffness hardening

## Abstract

With advancements in seismic isolation and damping technology, high-damping rubber (HDR) bearings are now widely used. However, significant gaps remain in HDR-analysis model research, with few studies integrating multiple factors, the Mullins effect, and stiffness hardening for more accurate practical predictions. This study classifies the effective behavior of HDR and examines the stress–strain relationships of different behavioral types using more appropriate equations. Mathematical models were established based on pseudo-elasticity theory, which is an extension of continuum mechanics. Subsequently, parameter functions were developed through parameter determination tests and regression analysis, leading to the completion of the pseudo-elastic model for HDR. Finally, the model’s effectiveness was validated through validation tests. This study finds that behavior classification effectively examines phenomenological-based HDR stress–strain relationships, as distinct behavioral patterns are not adequately captured by a single approach. Incorporating tests to functionalize material parameters complements theoretical models. Additionally, accurately explaining HDR behavior requires considering the Mullins effect and stiffness hardening, influenced by the coupled effects of temperature, strain amplitude, and compressive stress. Consequently, this HDR pseudo-elastic model offers a comprehensive explanation of HDR behavior, including the Mullins effect and stiffness hardening, under various influencing factors based on clear mechanical principles and explicit computational procedures.

## 1. Introduction

In recent years, the development of seismic technology has received increasing attention. The following major earthquakes such as the 1995 Kobe earthquake in Japan [1], the 2004 Sumatra earthquake in Indonesia [2], the 2008 Wenchuan earthquake in China [3], and the 2011 Tōhoku earthquake in Japan [4] have deepened people’s understanding of the catastrophic damage earthquakes can cause, both domestically and internationally. In the wake of the Wenchuan earthquake, many scholars have analyzed and discussed seismic technology for bridges in China [5,6].

Traditional seismic design methods rely on increasing the strength and stiffness of structural components to withstand earthquakes. However, these methods often underperform during actual events. As a result, seismic design research has shifted towards approaches that reduce seismic responses and dissipate energy [7]. Isolation and damping technologies are key among these new methods, significantly protecting structures from damage and easing post-earthquake repairs. High-damping rubber (HDR) bearings are a crucial component of these technologies, offering non-pollution and high-damping benefits. HDR bearings have shown excellent performance in both practical applications and theoretical studies, leading to their widespread use [8,9].

Temperature, shear-strain amplitude, compressive stress, and shear frequency are four common factors affecting the performance of HDR bearings. Actually, HDR is very sensitive to temperature, because the reinforcing agent of HDR contains more carbon black, white-carbon black or graphite, and these fillers are sensitive to temperature changes. The horizontal shear stiffness of the HDR bearing at 0 °C and 23 °C may be nearly twice as much for the latter, and the horizontal shear deformation at −20 °C is very difficult [10]. In tropical regions, it is common for bridge bearings to operate at temperatures above 30 °C during the summer. However, Chen et al. found that within the temperature range of −20 to 40 °C, the area of the hysteresis loop and the equivalent damping ratio of the HDR bearings significantly decreased with increasing temperature [11]. Ignoring temperature effects in cases with large temperature differences can lead to significant discrepancies between simulated calculations and actual test results [12,13]. Additionally, the mechanical behavior of HDR bearings is highly sensitive to shear-strain amplitude. As strain amplitude increases, the compressed area in the core of HDR bearings decreases, leading to reduced horizontal stiffness in areas farther from the core. Markou A. A. and colleagues recorded in their book *Dynamic Response of Infrastructure to Environmentally Induced Loads* that during displacement-controlled harmonic tests for the Solarino project, the hysteresis loop of HDR bearings appeared elliptical at small-strain amplitudes. However, as strain amplitude increased, the hysteresis loop evolved into a pronounced crescent shape, indicating a stiffness-hardening phenomenon [14]. The vertical compressive stress has a non-negligible effect on the energy dissipation capacity of the HDR bearing. With the increase in the compressive stress, the stiffness-hardening phenomenon caused by large shear strain becomes more significant. The hysteretic curve gradually changes from a spindle shape to an S shape, and the energy dissipation capacity increases approximately linearly [15,16]. Markou A. A. et al. [17] conducted shear-strain and frequency correlation tests on HDR bearings with constant compressive stress and shear strains of 120% and 200%, respectively, across frequencies ranging from 0.006 Hz to 0.83 Hz. They indicated that the mechanical properties of HDR bearings may have little relation to frequency.

Stress softening is also an important phenomenon in the study of HDR that cannot be overlooked. It can be summarized as follows: when HDR undergoes a loading–unloading–reloading cycle within a range smaller than the maximum historical-strain amplitude, the unloading stress and reloading stress are much lower than the initial loading stress [18]. This phenomenon, known as the Mullins effect, was first introduced and experimentally studied by Holt, W. L., in 1932 [19]. Since the beginning of the 21st century, some researchers have further investigated the Mullins effect, focusing mainly on developing numerical models for the Mullins effect and exploring the impact of working conditions on it [20,21].

The analytical model describes the stress–strain relationship of a specific material, making its development fundamental for studying the material’s mechanical properties. Currently, analytical models for HDR are primarily divided into four categories: purely mathematical fitting models [22,23], machine learning models [24], molecular-chain network models [25], and phenomenological models [26]. Earlier HDR models often relied on purely mathematical fitting models that used expressions weakly connected to mechanical principles, resulting in research findings confined to limited experimental data. HDR models developed using machine learning leverage extensive experimental data to train the model, bypassing mechanical principles and fundamental assumptions. However, the complexity of these training models means that developers often do not fully understand their underlying computational principles. The molecular-chain network model based on thermodynamic statistical theory effectively captures macroscopic material behavior through statistical distributions, but its reliance on oversimplified assumptions and accurate input parameters limits its applicability, to some extent. Phenomenological models, which are chosen for this study, are supported by principles of continuum mechanics and combined with a substantial amount of experimental data and clear statistical methods, enhancing their generalizability. However, one of the main drawbacks of these models is their reliance on fundamental assumptions. Although many models exist to describe HDR’s effective behavior, the model proposed in this study differs from these existing models, as most of them focus only on loading and unloading processes or single influencing factors, lacking a comprehensive approach that considers the interactions among loading, unloading, the Mullins effect, stiffness hardening, and multiple coupling influences [24,27].

The Ogden strain-energy function is widely used in studying the analytical models of rubber materials and the Mullins effect. Ogden R. W. [28] proposed the Ogden strain-energy function based on continuum mechanics. This strain-energy function depends only on the final state of strain and is independent of strain history, using the principal stretch ratios as variables. There are two basic assumptions: (1) the rubber is an isotropic material, and (2) the rubber is an incompressible material. Pseudo-elasticity theory is a new approach proposed by Ogden and Roxburgh [29] for studying the Mullins effect and the unloading process in rubber, which quantitatively analyses changes in strain energy by considering cumulative damage, serving as an extension of continuum mechanics.

However, there are still many gaps in the research field of analytical models for HDR that need to be further investigated. Among the current research findings on HDR models, few integrate the coupled effects of multiple factors and reflect the Mullins effect. This paper will investigate the stress-strain responses of HDR based on pseudo-elasticity theory, combined with multi-factor compression–shear tests. A multi-factor compression–shear pseudo-elastic model for HDR will be proposed, which not only considers the coupled effects of temperature, compressive stress, and shear-strain amplitude, but also incorporates the Mullins effect and stiffness hardening. Furthermore, this model is phenomenological, with each step having a strict and clear mechanical basis and explicit computational procedures, fundamentally ensuring its universality and the feasibility for future development.

## 2. Test Methods

To accurately simulate the working conditions of HDR bearings, this study performed compression–shear cyclic loading tests that considered the combined effects of varying temperatures, compressive stresses, and shear-strain amplitudes. The design of the test specimens was based on GB 20688.2-2006 [30], while the testing methods referenced GB 20688.1-2007 [31], as well as the works of Li [24] and Dorfmann [20].

### 2.1. Test Set-Up

**Figure 1:**
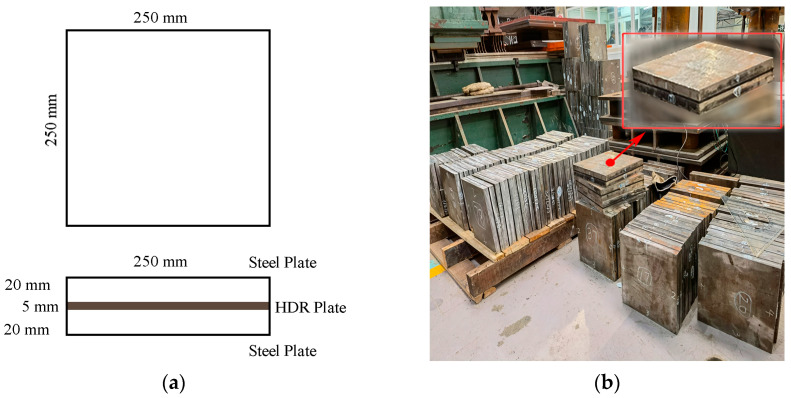
High-damping rubber (HDR) test specimens: (**a**) the schematic diagram of HDR test specimens; (**b**) the actual image of HDR test specimens.

**Figure 2:**
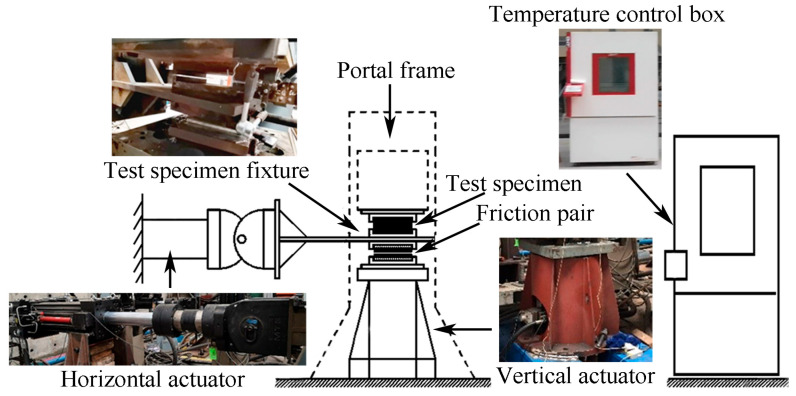
Test devices.

The HDR specimens used in this study have a total dimension of 250 mm×250 mm×25 mm, with the top and bottom plates made of Q235 steel plates sized 250 mm×250 mm×20 mm, and a central layer of HDR with a dimension of 250 mm×250 mm×5 mm, which meets the requirements of the first form factor S_1_ and the second form factor S_2_ in GB 20688.2-2006. The schematic and actual images of the test specimens are shown in Figure 1. Moreover, the configuration of the devices is illustrated in Figure 2. A test chamber with alternating high and low temperatures (measurement accuracy: ±0.5 K), manufactured by DOAHO Test Equipment Factory in Shanghai, China, was used for temperature control. Horizontal loading was performed with a 1000 kN actuator from MTS Systems Corporation (measurement accuracy: ±1% of the load reading, ±1% of the displacement reading), Eden Prairie, MN, USA, while vertical loading was managed with a 2000 kN actuator from Beijing Fluid Control System Corp (measurement accuracy: ±1% of the load reading), Shanghai, China. In addition, the FlexTest GT control system developed by MTS Systems Corporation of the United States was used in this study.

### 2.2. Test Procedure and Test Cases

To minimize the potential specificity and limitations of the test results, HDR specimens used in this study were sourced from two different production batches, and at least three specimens were tested under each test case.

Since this study involves temperature variations, a preliminary mechanical-performance stability test was conducted on HDR specimens to determine an adequate equilibration time. For rigor, all specimens in this study were equilibrated for over 10 h before testing.

**Figure 3:**
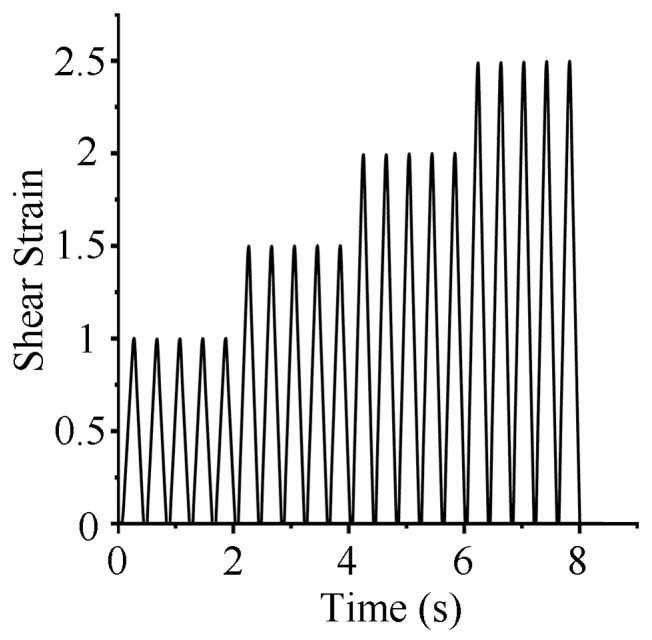
The loading mode of the cyclic loading test: multistage triangular wave.

The test utilized multi-level triangular waves for displacement loading. At four different levels of shear-strain amplitude, each amplitude level underwent five cycles before transitioning to the next strain level. The test cases were designed to include combinations of different temperatures and compressive stresses. Since the study found that shear frequency has a minor effect on the mechanical properties of HDR, similar to the conclusions of Markou A. A. et al. [17], shear frequency was not considered a factor in this research. A loading frequency of 0.4 Hz was used for all cases. Figure 3 depicts the loading mode of the cyclic loading test: the multistage triangular wave. This study includes two types of tests: parameter determination tests for developing the pseudo-elastic model and validation tests for verifying the effectiveness of the pseudo-elastic model. All test cases are listed in Table 1 and Table 2.

## 3. Development of the Pseudo-Elastic Model

Since the development of the model proposed in this study is primarily based on pseudo-elasticity theory, there are two basic assumptions: (1) the rubber is an isotropic material, and (2) the rubber is an incompressible material. In addition, this study will develop the model under the following boundary conditions: (1) temperature: 253.15 K to 313.15 K, (2) shear strain: 0–250%, and (3) compressive stress: 6–10 MPa. Furthermore, this study will consider loading, unloading, the Mullins effect, and stiffness-hardening phenomena through a behavioral classification approach. It will also directly address the effects of compressive stress through the development of the mechanics-based model and further consider the impacts of temperature and strain amplitude by combining test data and regression analysis.

### 3.1. Classification of Effective Behavior

**Figure 4:**
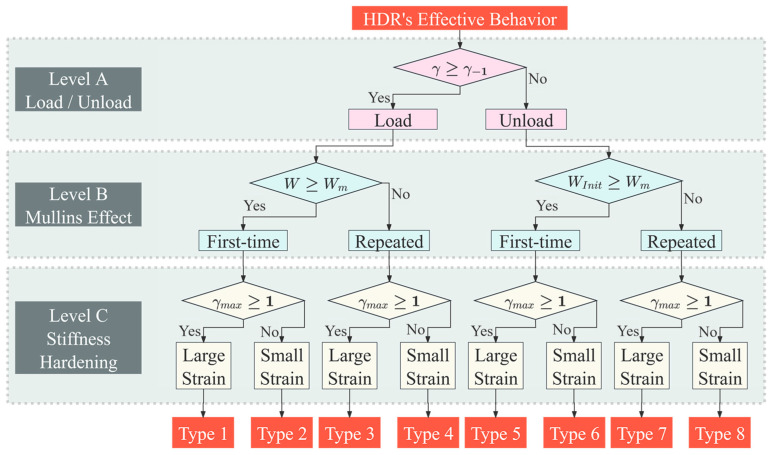
Classification of effective behavior of HDR.

HDR exhibits significant variations in effective behavior under different loading stages, to the extent that a single mechanical theory may not be adequate for its explanation. Therefore, to provide a comprehensive and accurate explanation of HDR’s effective behavior across various loading stages, this study has established three judgment levels for classifying HDR’s effective behavior. Specifically, as illustrated in Figure 4:

- Level A classifies effective behavior as either loading or unloading, by comparing the shear strain at the previous moment $\gamma_{-1}$ with the shear strain at the current moment γ;
- Level B further categorizes the effective behavior as either first-time or repeated loading/unloading at the current strain amplitude, by examining the relationship between the current strain energy W or the initial strain energy of the current unloading behavior $W_{Init}$ and the historical maximum strain energy $W_{m}$, in preparation for analyzing the Mullins effect;
- Level C further divides the effective behavior into large strain and small strain, based on whether the shear-strain amplitude $\gamma_{max}$ exceeds 100%, in preparation for analyzing stiffness hardening.

The resulting eight behavior types cover all possible behaviors of HDR.

### 3.2. Model Development Process

This study employed two different methods to comprehensively explain the various types of HDR’s effective behavior and the impact of multiple factors on it.

Firstly, specific issues in developing the mathematical model were addressed using the theoretical foundation of mechanics. Compressive stress effects are also incorporated directly into the model.

Secondly, since mechanical theories alone cannot resolve all issues, test data analysis is crucial. This analysis reveals how material parameters are influenced by temperature and shear-strain amplitude under different behaviors.

**Figure 5:**
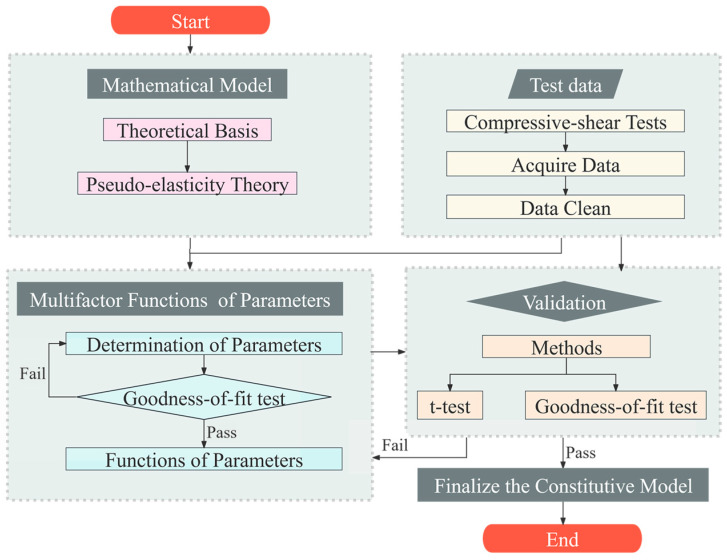
The process of developing the proposed pseudo-elastic model.

Figure 5 illustrates the process:

1. Mathematical models for loading and unloading behaviors are developed based on pseudo-elasticity theory, with cumulative damage considered only during the unloading phase, to explain energy changes.
2. Compression–shear cyclic loading tests are conducted under various conditions to acquire data and clean it.
3. The mathematical models are integrated with parameter-determination test data to establish material parameter values for each test case and identify the variation patterns of these parameters with changing working conditions, namely, the functions of the parameters.
4. The model’s effectiveness is validated by comparing its results with those from validation tests. If the model’s calculations align well with the validation tests, the pseudo-elastic model is finalized.

### 3.3. Loading-Behavior Model

The application of the strain-energy function is directly related to the strain state of the material being studied. HDR bearings, used in bridge-isolation applications, operate under a compression–shear-strain state. Therefore, it is necessary to apply the strain-energy function based on the compression–shear-strain state for deriving the model. This study uses the Ogden strain-energy function [28].

Based on the three directions of the coordinate system, the coordinates of the initial unstrained state are defined as $X_{i}\left(i=1,2,3\right)$, while the coordinates of the compression–shear-strain state are defined as $x_{1i}\left(i=1,2,3\right)$.

Let a be the principal length ratio in the compressive axial direction, and let γ be the shear strain a=1−ε=1−N/E; ε is the compressive strain, N is the compressive stress, and E is the elastic modulus:

(1) $$\left\{\begin{array}{l} x_{11}=a^{-\frac{1}{2}}X_{1}+{a\gamma X}_{3}\\ x_{12}=a^{-\frac{1}{2}}X_{2}\\ x_{13}={aX}_{3} \end{array}\right.$$

Thus, the deformation gradient tensor F and the right Cauchy–Green tensor C can be obtained as follows:

(2) $$F=\left[\begin{array}{ccc} a^{-\frac{1}{2}} & 0 & a\gamma \\ 0 & a^{-\frac{1}{2}} & 0\\ 0 & 0 & a \end{array}\right]$$

(3) $$C=\left[\begin{array}{ccc} a^{-1} & 0 & a^{\;\frac{1}{2}}\gamma \\ 0 & a^{-1} & 0\\ a^{\;\frac{1}{2}}\gamma & 0 & a^{2}\left(\gamma^{2}+1\right) \end{array}\right]$$

Since the eigenvalues $\beta_{\alpha }\left(\alpha =1,2,3\right)$ of the right Cauchy–Green tensor C are related to the principal length ratios under the compression–shear-strain state $\lambda_{L_{\alpha }}\left(\alpha =1,2,3\right)$ by $\lambda_{L_{\alpha }}=\sqrt{\beta_{\alpha }}$ and the eigenvectors $n_{\alpha }^{c}\left(\alpha =1,2,3\right)$ are related to the principal direction vectors $L_{\alpha }\left(\alpha =1,2,3\right)$ by $L_{\alpha }=n_{\alpha }^{c}$, the following can be obtained:

(4) $$\left\{\begin{array}{l} \lambda_{L_{1}}=\sqrt{\beta_{1}}=\sqrt{\frac{a^{2}\left(\gamma^{2}+1\right)+a^{-1}-\sqrt{{\left[a^{2}\left(\gamma^{2}+1\right)-a^{-1}\right]}^{2}+4a}}{2}}\\ \lambda_{L_{2}}=\sqrt{\beta_{2}}=a^{-\frac{1}{2}}\\ \lambda_{L_{3}}=\sqrt{\beta_{3}}=\sqrt{\frac{a^{2}\left(\gamma^{2}+1\right)+a^{-1}+\sqrt{{\left[a^{2}\left(\gamma^{2}+1\right)-a^{-1}\right]}^{2}+4a}}{2}} \end{array}\right.$$

(5) $$\left\{\begin{array}{l} L_{1}=n_{1}^{c}={\left(\frac{-2a^{\frac{1}{2}}\gamma }{a^{-1}-a^{2}\left(\gamma^{2}+1\right)+\sqrt{{\left[a^{2}\left(\gamma^{2}+1\right)-a^{-1}\right]}^{2}+4a}}\;,0\;,1\right)}^{T}\\ L_{2}=n_{2}^{c}={\left(0\;,1\;,0\right)}^{T}\\ L_{3}=n_{3}^{c}={\left(\frac{-2a^{\frac{1}{2}}\gamma }{a^{-1}-a^{2}\left(\gamma^{2}+1\right)-\sqrt{{\left[a^{2}\left(\gamma^{2}+1\right)-a^{-1}\right]}^{2}+4a}}\;,0\;,1\right)}^{T} \end{array}\right.$$

The Ogden strain-energy function $W_{Ogden}$, expressed in terms of the principal length ratios can be written as

(6) $$W_{Ogden}=\sum_{i=1}^{3}\frac{\mu_{i}}{\alpha_{i}}\left(\lambda_{L1}^{\alpha_{i}}+\lambda_{L2}^{\alpha_{i}}+\lambda_{L3}^{\alpha_{i}}-3\right)$$

The Cauchy stress in the principal directions $\sigma_{\alpha }$ can be expressed as follows, where P is the hydrostatic pressure:

(7) $$\sigma_{\alpha }=\lambda_{L\alpha }\frac{\partial W\left(\lambda_{L1}\;,\lambda_{L2}\;,\lambda_{L3}\right)}{\partial \lambda_{L\alpha }}-P,\left(\alpha =1,2,3\right)$$

Let θ denote the angle of rotation of the principal axes during the compression–shear-strain process. Then, the shear stress τ parallel to the shear plane can be written as

(8) $$\tau =\frac{1}{2}\left(\sigma_{1}-\sigma_{3}\right)\sin2\theta =\frac{1}{2}\sum_{i=1}^{3}\mu_{i}\left(\lambda_{L1}^{\alpha_{i}}-\lambda_{L3}^{\alpha_{i}}\right)\sin2\theta$$

In the above equation, $\mu_{i}$ and $\alpha_{i}\left(i=1,2,3\right)$ are material parameters that need to be determined through parameter determination tests. Furthermore, sin⁡2θ is determined from the principal direction vectors. For simplicity, two parameters A and B are introduced. The principal direction vector $L_{1}$ from Equation (5) is used to obtain the following:

(9) $$\sin2\theta =\frac{A}{\sqrt{A^{2}+B^{2}}}$$

where

(10) $$A=4a^{\frac{1}{2}}\gamma \left\{-\sqrt{{\left[a^{2}\left(\gamma^{2}+1\right)-a^{-1}\right]}^{2}+4a}+a^{2}\left(\gamma^{2}+1\right)-a^{-1}\right\}\;B=4a\gamma^{2}-{\left\{a^{-1}-a^{2}\left(\gamma^{2}+1\right)+\sqrt{{\left[a^{2}\left(\gamma^{2}+1\right)-a^{-1}\right]}^{2}+4a}\right\}}^{2}$$

Finally, the loading-behavior model is summarized as follows:

(11) $$\tau =\frac{1}{2}\frac{A}{\sqrt{A^{2}+B^{2}}}\sum_{i=1}^{3}\mu_{i}\left(\lambda_{L1}^{\alpha_{i}}-\lambda_{L3}^{\alpha_{i}}\right)$$

### 3.4. Unloading-Behavior Model

This study modifies the strain-energy function for loading behavior by introducing a cumulative damage function based on pseudo-elasticity theory [29], enabling it to account for changes in strain energy. Consequently, an unloading-behavior model is derived.

At this stage, Equation (6) is revised, with the modified strain-energy function W expressed in terms of the principal length ratio and the continuous damage variable, where $\phi \left(\eta \right)$ is the damage function and η is the continuous damage variable.

(12) $$\begin{array}{cc} W\left(\lambda_{L1}\;,\lambda_{L2}\;,\lambda_{L3}\;,\eta \right) & =\eta W_{Ogden}\left(\lambda_{L1}\;,\lambda_{L2}\;,\lambda_{L3}\right)+\phi \left(\eta \right)\\ & =\eta \sum_{i=1}^{3}\frac{\mu_{i}}{\alpha_{i}}\left(\lambda_{L1}^{\alpha_{i}}+\lambda_{L2}^{\alpha_{i}}+\lambda_{L3}^{\alpha_{i}}-3\right)+\phi \left(\eta \right) \end{array}$$

The Cauchy stress in the principal direction of unloading behavior $\sigma_{u\alpha }$ can be expressed as

(13) $$\begin{array}{cc} \sigma_{u\alpha } & =\lambda_{L\alpha }\frac{\partial W\left(\lambda_{L1}\;,\lambda_{L2}\;,\lambda_{L3}\;,\eta \right)}{\partial \lambda_{L\alpha }}-P\\ & =\lambda_{L\alpha }\frac{\partial \left[{\eta W}_{0}\left(\lambda_{L1}\;,\lambda_{L2}\;,\lambda_{L3}\right)+\phi \left(\eta \right)\right]}{\partial \lambda_{L\alpha }}-P,\left(\alpha =1\;,2\;,3\right) \end{array}$$

In unloading behavior, the shear stress $\tau_{u}$ parallel to the shear plane can be expressed as

(14) $$\tau_{u}=\frac{1}{2}\left(\sigma_{u1}-\sigma_{u3}\right)\sin2\theta =\eta \left[\frac{1}{2}\sum_{i=1}^{3}\mu_{i}\left(\lambda_{L1}^{\alpha_{i}}-\lambda_{L3}^{\alpha_{i}}\right)\sin2\theta \right]$$

By substituting the equation for the continuous damage variable η [29] and combining it with Equation (9), the following can be obtained:

(15) $$\tau_{u}=\frac{1}{2}\frac{A}{\sqrt{A^{2}+B^{2}}}\left\{1-\frac{1}{r}erf\left[\frac{1}{m}\left(W_{m}-W_{Ogden}\left(\lambda_{L1}\;,\lambda_{L2}\;,\lambda_{L3}\right)\right)\right]\right\}\times \sum_{i=1}^{3}\mu_{i}\left(\lambda_{L1}^{\alpha_{i}}-\lambda_{L3}^{\alpha_{i}}\right)$$

Finally, by adding a term $De^{-\gamma }$ to the above expression to account for the residual stress at the end of unloading, the ultimate unloading-behavior model is obtained:

(16) $$\tau_{u}=\frac{1}{2}\frac{A}{\sqrt{A^{2}+B^{2}}}\left\{1-\frac{1}{r}erf\left[\frac{1}{m}\left(W_{m}-W_{Ogden}\left(\lambda_{L1}\;,\lambda_{L2}\;,\lambda_{L3}\right)\right)\right]\right\}\times \sum_{i=1}^{3}\mu_{i}\left(\lambda_{L1}^{\alpha_{i}}-\lambda_{L3}^{\alpha_{i}}\right)+De^{-\gamma }$$

where $erf\left(x\right)=\frac{2}{\sqrt{\pi }}\int_{0}^{x}e^{-y^{2}}dy$, $W_{m}=\underset{\left[t_{0},t\right]}{\max}\left(W_{Ogden}\;,W\right)$, r, m and D are material parameters that need to be determined through parameter identification tests. In the unloading-behavior model, $\mu_{i}$ and $\alpha_{i}$, where $\left(i=1,\;2,\;3\right)$, are inherited from the loading behavior connected before the unloading behavior is triggered, and do not need to be determined again.

## 4. Results

By developing both loading- and unloading-behavior models based on mechanical theories, this study directly classifies and considers the HDR’s loading and unloading behaviors, theoretically. The models also reflect the influence of compressive stress on the horizontal shear stress of HDR rubber plates. However, material parameters must be determined in conjunction with parameter determination test data. Additionally, exploring the functional relationships of the material parameters can serve as a complement to the mechanical principles, enabling the final pseudo-elastic model to further explain the Mullins effect and stiffness-hardening effect, as well as to reflect the impact of temperature and shear-strain amplitude on HDR shear stress. Furthermore, after the pseudo-elastic model is established, it is essential to conduct validation tests to assess its effectiveness, in order to finalize the model.

### 4.1. Multifactor Functions of Parameters

**Figure 6:**
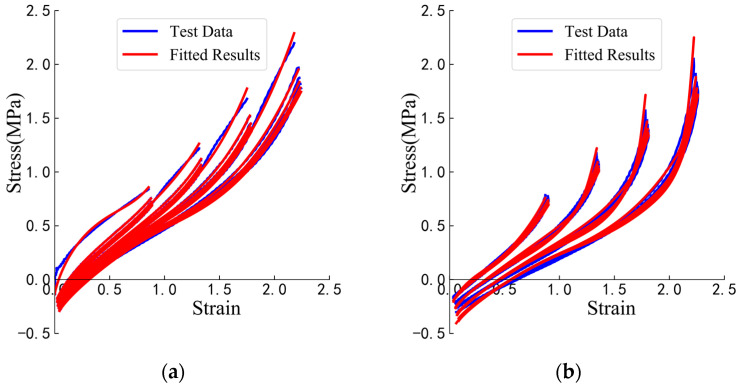
Examples of curve-fitting results: (**a**) loading-behavior results; (**b**) unloading-behavior results.

Curve fitting is performed on the parameter-determination test data, using both the loading- and unloading-behavior models. The fit quality is assessed using the multiple R-squared value, with values closer to 1 indicating a better fit [32,33]. The parameter determination tests in this study involve numerous cases. For example, under the test case (temperature: 313.15 K, compressive stress: 10 MPa, shear-strain amplitudes: 100%–150%–200%–250%), as shown in Figure 6, the multiple R-squared values for each loading and unloading behavior are all greater than 0.99. This also indicates that the two mathematical models derived in this study are reasonable.

Each fitting curve for the cases of the parameter-determination tests will yield the corresponding material parameters, $\mu_{i}$ and $\alpha_{i}$, where $\left(i=1,2,3\right)$ for the loading behavior and r, m and D for the unloading behavior. When the multiple R-squared value exceeds 0.99, it can be considered that the fitting curve sufficiently matches the test data, and the material parameters are then finalized. Since 20 cycles are required under each test condition, the curve-fitting results, as shown in Figure 6, produce 20 sets of material parameters for the loading behavior and 20 sets for the unloading behavior.

This study employs regression analysis to uncover the relationships between material parameters and develop multifactor functions of parameters. This approach enhances the purely theoretical mathematical models for loading and unloading behavior by further incorporating the effects of temperature and strain amplitude. Additionally, it provides a more specific correspondence with HDR’s effective behavior, as categorized in Figure 4, to further explain the Mullins effect and stiffness-hardening effect.

**Figure 7:**
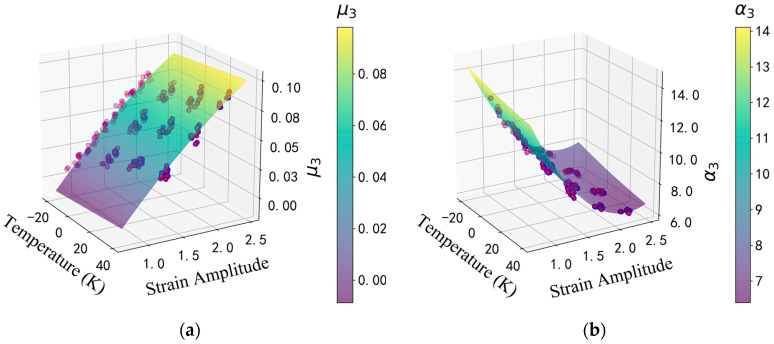
Summary examples of regression analysis results: (**a**) the regression analysis result of the material parameters $\mu_{3}$ from the third type of HDR’s effective behavior; (**b**) the regression analysis results of the material parameters $\alpha_{3}$ from the third type of HDR’s effective behavior.

Excluding the compressive stress already directly reflected during the purely theoretical derivation phase, this study uses temperature and shear-strain amplitude as two factors and performs regression analysis on the material parameters based on the Two-factor Response Surface Model [34]:

(17) $$y(T,\gamma_{max})=C_{00}+C_{10}T+C_{20}T^{2}+{C_{01}\gamma_{max}+C}_{02}\gamma_{max}^{2}+C_{11}T\gamma_{max}+\epsilon$$

where y represents the material parameters, T is the temperature, $\gamma_{max}$ is the shear-strain amplitude, $C_{ij}$ is where (i, j=0,1,2) are the regression coefficients and ϵ is the random error. This study uses *t*-tests to assess the significance of regression coefficients, specifically to determine whether the explanatory variables in the regression model have a significant effect on the dependent variable. Each F-statistic is computed, and its associated *p*-value indicates the probability that the value of the regression coefficient could be due to random chance [32]. A threshold value of 0.05 (5%) is used in this study to filter out variables that make a significant contribution to the regression model, retaining only those regression coefficients with *p*-values less than 0.05. In addition, the R-squared value for each regression model is calculated, with R-squared values set to be greater than 0.985 to ensure the overall significance of the regression models. Using the regression analysis results of the material parameters $\mu_{3}$ and $\alpha_{3}$, from the third type of HDR’s effective behavior in Figure 4 as an example, the results are summarized in Figure 7. The regression analysis results for all multifactor functions of parameters corresponding to the eight types of HDR’s effective behavior will be presented in detail, next.

#### 4.1.1. Functions of Loading Parameters

As illustrated in Figure 4 above, HDR loading behaviors are further divided into four types, based on the Mullins effect and stiffness-hardening effect: Type 1–Type 4. Each effective behavior has specific functions for parameters $\mu_{i}(T,\;\gamma_{max})$ and $\alpha_{i}(T,\;\gamma_{max})$, where $\left(i=1,2,3\right),$ which need to be determined. Although constants ($\mu_{1}=35$, $\mu_{2}=15$ and $\alpha_{2}=-0.001$) remain unchanged across behavior types, the functions for parameters $\mu_{3}(T,\;\gamma_{max}),\;\alpha_{1}(T,\;\gamma_{max})\;and\;\alpha_{3}(T,\;\gamma_{max})\;$ are detailed in Table 3, where the shear strain at the previous moment $\gamma_{-1}$, the shear strain at the current moment γ, the current strain energy W, the historical maximum strain energy $W_{m}$ and the shear-strain amplitude $\gamma_{max}$ have the same meaning as described earlier.

#### 4.1.2. Functions of Unloading Parameters

As illustrated in Figure 4 above, HDR unloading behaviors are also further divided into four types, based on the Mullins effect and stiffness-hardening effect: Type 5–Type 8. Parameters $\mu_{i}$ and $\alpha_{i}$, are those where $\left(i=1,2,3\right)$ for each unloading behavior are directly inherited from the loading behavior that triggered the unloading. The functions of parameters that need to be determined are the material parameters $r(T,\;\gamma_{max})$, $m(T,\;\gamma_{max})\;and\;D(T,\;\gamma_{max})$, corresponding to each type of unloading behavior. The results are detailed in Table 4, where $W_{Init}$ is the initial strain energy of the current unloading behavior, and other symbols are as defined earlier.

### 4.2. Validation of the Pseudo-Elastic Model

**Figure 8:**
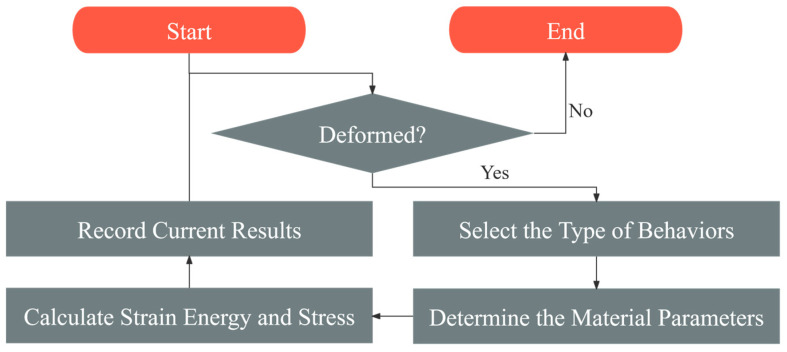
The calculation flow of the HDR pseudo-elastic model proposed in this paper.

After determining all the functions for the material parameters, the pseudo-elastic model is complete. Finally, the validity of the pseudo-elastic model will be verified by comparing the model’s computational results with the validation test data. In particular, the calculation flow of the multi-factor HDR pseudo-elastic model proposed in this paper is shown in Figure 8. When the HDR plate undergoes shear deformation, first assess the deformation behavior and classify it into one of the eight types. Then, use the material parameter functions corresponding to that type to compute the parameter values. Next, calculate the strain energy and shear stress at the current time, based on the loading- or unloading-behavior model, and record the results. If deformation continues, use the current results for the next time step, repeating this process until deformation stops.

**Figure 9:**
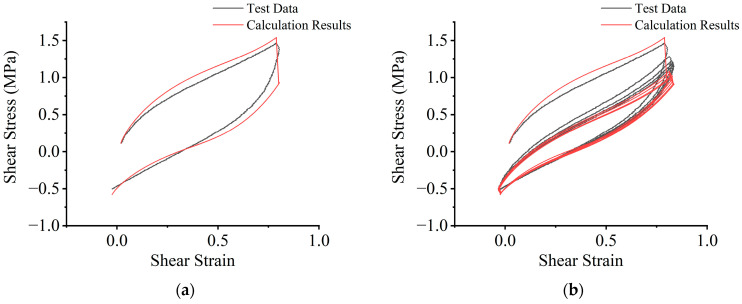

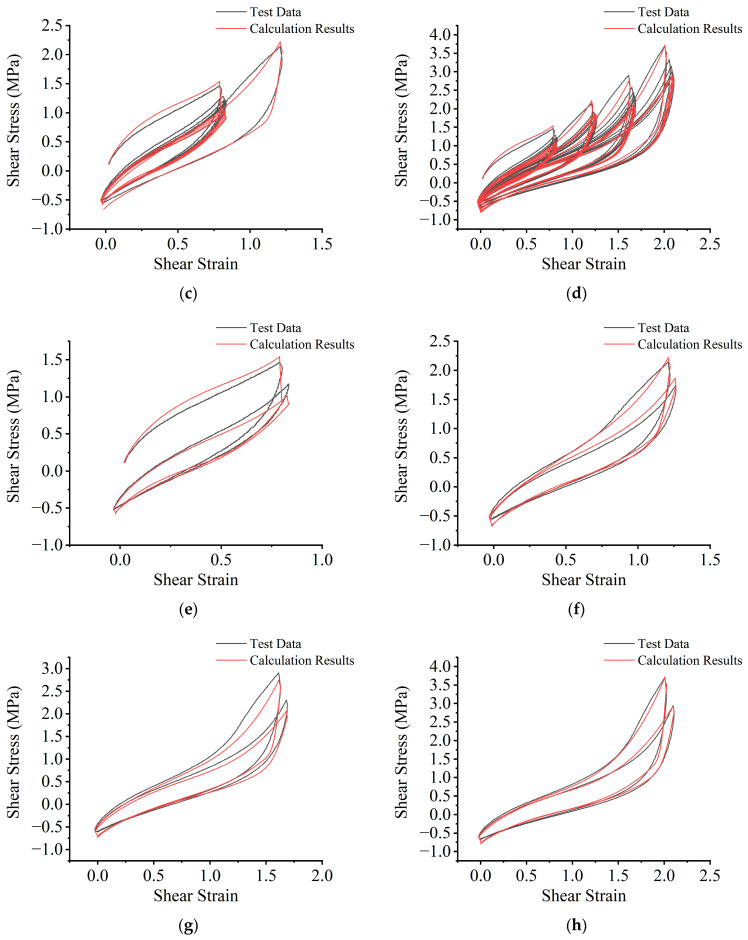
Examples of the pseudo-elastic model validation results: (**a**) HDR’s effective behavior types 2 and 6; (**b**) HDR’s effective behavior types 2, 4, 6 and 8; (**c**) HDR’s effective behavior types 1, 2, 4–6 and 8; (**d**) HDR’s effective behavior types 1–8; (**e**) comparison of test data and calculated results for the first loading and fifth loading at a strain amplitude of 90%; (**f**) comparison of test data and calculated results for the first loading and fifth loading at a strain amplitude of 130%; (**g**) comparison of test data and calculated results for the first loading and fifth loading at a strain amplitude of 170%; (**h**) comparison of test data and calculated results for the first loading and fifth loading at a strain amplitude of 210%.

Figure 9 provides an example with validation test cases of temperature at 278.15 K, compressive stress at 9.5 MPa, and four levels of shear-strain amplitude of 90%, 130%, 170%, and 210%. The R-squared value between the test data and the calculation results of the pseudo-elastic model is greater than 0.98. To visually demonstrate that the pseudo-elastic model proposed in this study can account for all eight effective behaviors of HDR under compression and shear deformation, including loading/unloading, Mullins effect, and stiffness-hardening effect, Figure 9a–d is divided into four subplots for detailed presentation. To further illustrate the validity of the proposed pseudo-elastic model and the application of the behavior classification method in this study, Figure 9e–h compare the test data and calculated results for both the first and fifth loading cycles (the latter considered stabilized after the Mullins effect) at shear-strain amplitudes of 90%, 130%, 170%, and 210%, respectively. Additionally, the R-squared values corresponding to the above cycles and all data are listed individually in Table 5.

Furthermore, as shown in Figure 9, there is a difference of over 20% in the maximum stress values between the first cycle at the same strain level and the stabilized fifth cycle after experiencing the Mullins effect. The phenomenon of stiffness hardening gradually becomes more apparent when the shear strain exceeds approximately 100%. Figure 7 also indicates that temperature and strain amplitude have a significant impact on the values of material parameters. Compressive stress has been directly integrated into the model-building process. Therefore, it is essential to comprehensively consider these factors for accurate predictions of HDR’s effective behavior.

## 5. Conclusions

This study primarily focuses on the classification of effective behaviors of HDR under compression and shear conditions with multiple factors, the development of mathematical models for loading and unloading behaviors, the determination of multifactor functions of material parameters, and the finalization of the pseudo-elastic model. The following conclusions were drawn:

1. Classifying effective behaviors is highly effective for phenomenological-based HDR model research methods. The patterns of parameters corresponding to different types of effective behaviors show significant differences, and it may not even be appropriate to explain them using the same equation.
2. Further integration with test data allows for a more specific explanation of the Mullins effect, stiffness-hardening effect, and the impacts of temperature and shear-strain amplitude through the material parameter functions.
3. The Mullins effect and stiffness-hardening effect are inevitable material behaviors in HDR applications, with temperature, shear-strain amplitude, and compressive stress being the most common influencing factors. Failure to consider these issues simultaneously may lead to significant calculation errors.
4. The pseudo-elastic model for HDR proposed in this study can effectively predict the behavior of HDR under compression–shear conditions. The R-squared values obtained from the validation test data compared to the calculated results of the model are greater than 0.98.

However, there are still many directions to explore in accurately predicting the effective behavior of HDR. For example, HDR can exhibit noticeable crystallization at lower temperatures, which may lead to distinctly different behavior classifications, necessitating the exploration of the possibility of describing it using other mechanical theories. Additionally, it is meaningful to broaden the scope of the coupling effects of influencing factors, such as incorporating frequency dependence, as this would make theoretical research more aligned with practical applications.

## Figures and Tables

**Table 1:** Parameter-determination test cases.

Influencing Factor	Values			
Temperature	253.15 K	273.15 K	293.15 K	313.15 K
Compressive Stress	6 MPa	8 MPa		10 MPa
Shear-Strain Amplitude	100%	150%	200%	250%

**Table 2:** Validation test cases.

Influencing Factor	Values			
Temperature	278.15 K		288.15 K	
Compressive Stress	6.5 MPa		9.5 MPa	
Shear-Strain Amplitude	90%	130%	170%	210%

**Table 3:** Multifactor parameter functions for High-damping rubber (HDR) loading behaviors.

HDR Behaviors *	Parameters	Functions
Type 1: $\gamma \geq \gamma_{-1}$$,\;W\geq W_{m}$$,\;\gamma_{max}\geq 1$	$\alpha_{1}(T,\;\gamma_{max})$	$3.17\times 10^{-2}-2.78\times 10^{-4}T-1.34\times 10^{-2}\gamma_{max}+4.06\times 10^{-6}T^{2}+3.61\times 10^{-3}\gamma_{max}^{2}$
	$\mu_{3}(T,\;\gamma_{max})$	$1.23\times 10^{-2}+9.46\times 10^{-5}T+2.21\times 10^{-2}\gamma_{max}+4.16\times 10^{-6}\gamma_{max}^{2}$
	$\alpha_{3}(T,\;\gamma_{max})$	$15.56-2.29\times 10^{-2}T-6.42\gamma_{max}+1.31\gamma_{max}^{2}$
Type 2: $\gamma \geq \gamma_{-1}$$,\;W\geq W_{m}$$,\;\gamma_{max}<1$	$\alpha_{1}(T,\;\gamma_{max})$	$0.14-0.13\gamma_{max}$
	$\mu_{3}(T,\;\gamma_{max})$	0.001
	$\alpha_{3}(T,\;\gamma_{max})$	$19.07-2.84\times 10^{-2}T-2.81\gamma_{max}^{2}$
Type 3: $\gamma \geq \gamma_{-1}$$,\;W<W_{m}$$,\;\gamma_{max}\geq 1$	$\alpha_{1}(T,\;\gamma_{max})$	$6.93\times 10^{-2}-3.55\times 10^{-4}T-4.96\times 10^{-2}\gamma_{max}+3.87\times 10^{-6}T^{2}+1.23\times 10^{-2}\gamma_{max}^{2}$
	$\mu_{3}(T,\;\gamma_{max})$	$-6.25\times 10^{-2}+1.11\times 10^{-4}T+8.19\times 10^{-2}\gamma_{max}-7.54\times 10^{-3}\gamma_{max}^{2}$
	$\alpha_{3}(T,\;\gamma_{max})$	$21.55-2.50\times 10^{-2}T-13.59\gamma_{max}+1.26\times 10^{-4}T^{2}+2.97\gamma_{max}^{2}$
Type 4: $\gamma \geq \gamma_{-1}$$,\;W<W_{m}$$,\;\gamma_{max}<1$	$\alpha_{1}(T,\;\gamma_{max})$	$6.02\times 10^{-2}-3.67\times 10^{-4}T+5.67\times 10^{-6}T^{2}-3.94\times 10^{-2}\gamma_{max}^{2}$
	$\mu_{3}(T,\;\gamma_{max})$	$4.48\times 10^{-3}+1.33\times 10^{-4}T$
	$\alpha_{3}(T,\;\gamma_{max})$	$22.46-0.1T-9.7\gamma_{max}+1.55\times 10^{-3}T^{2}$

* Detailed explanations can be found in Figure 4.

**Table 4:** Multifactor parameter functions for HDR unloading behaviors.

HDR Behaviors *	Parameters	Functions
Type 5: $\gamma <\gamma_{-1}$$,\;W\geq W_{m}$$,\;\gamma_{max}\geq 1$	$r(T,\;\gamma_{max})$	$1.8+1.51\times 10^{-2}T+0.61\gamma_{max}-0.28\gamma_{max}^{2}$
	$m(T,\;\gamma_{max})$	$10.51-9.45\gamma_{max}+7.72\times 10^{-4}T^{2}+2.76\gamma_{max}^{2}$
	$D(T,\;\gamma_{max})$	$-0.49+9.87\times 10^{-3}T+0.17\gamma_{max}-1.60\times 10^{-4}T^{2}-8.23\times 10^{-2}\gamma_{max}^{2}$
Type 6: $\gamma <\gamma_{-1}$$,\;W\geq W_{m}$$,\;\gamma_{max}<1$	$r(T,\;\gamma_{max})$	$-1.28+10.31\gamma_{max}-2.78\times 10^{-4}T^{2}-7.11\gamma_{max}^{2}$
	$m(T,\;\gamma_{max})$	$125.68+0.39T-286.92\gamma_{max}+5.97\times 10^{-3}T^{2}+190.94\gamma_{max}^{2}$
	$D(T,\;\gamma_{max})$	$-2.11+4.95\times 10^{-3}T+4.17\gamma_{max}-8.20\times 10^{-5}T^{2}-2.42\gamma_{max}^{2}$
Type 7: $\gamma <\gamma_{-1}$$,\;W<W_{m}$$,\;\gamma_{max}\geq 1$	$r(T,\;\gamma_{max})$	$2.73+1.88\times 10^{-2}T-2.83\times 10^{-4}T^{2}-0.15\gamma_{max}^{2}$
	$m(T,\;\gamma_{max})$	$23.91+5.40\times 10^{-2}T-19.8\gamma_{max}+2.03\times 10^{-3}T^{2}+5.12\times 10^{-2}\gamma_{max}^{2}$
	$D(T,\;\gamma_{max})$	$-0.31+8.40\times 10^{-3}T-1.30\times 10^{-4}T^{2}-3.76\times 10^{-2}\gamma_{max}^{2}$
Type 8: $\gamma <\gamma_{-1}$$,\;W<W_{m}$$,\;\gamma_{max}<1$	$r(T,\;\gamma_{max})$	$-11.86+37.99\gamma_{max}-2.72\times 10^{-4}T^{2}-24.95\gamma_{max}^{2}$
	$m(T,\;\gamma_{max})$	$147.61+0.6T-318.1\gamma_{max}+209.95\gamma_{max}^{2}$
	$D(T,\;\gamma_{max})$	$-2.58+4.58\times 10^{-3}T+5.41\gamma_{max}-6.79\times 10^{-5}T^{2}-3.21\gamma_{max}^{2}$

* Detailed explanations can be found in Figure 4.

**Table 5:** R-squared values between the test data and calculated results for the validation case.

Strain Amplitude	90%	130%	170%	210%
First loading cycle	0.96	0.99	0.97	0.99
Fifth loading cycle	0.98	0.98	0.99	0.99
All loading cycles	0.98			

## Data Availability

Data are contained within the article.
